# Confirmatory Factor Analysis of Three Versions of the Depression Anxiety Stress Scale (DASS-42, DASS-21, and DASS-12) in Polish Adults

**DOI:** 10.3389/fpsyt.2021.770532

**Published:** 2022-01-04

**Authors:** Marta Makara-Studzińska, Ernest Tyburski, Maciej Załuski, Katarzyna Adamczyk, Jacek Mesterhazy, Agnieszka Mesterhazy

**Affiliations:** ^1^Department of Health Psychology, Institute of Nursing and Midwifery, Jagiellonian University Medical College, Kraków, Poland; ^2^Department of Health Psychology, Pomeranian Medical University in Szczecin, Szczecin, Poland

**Keywords:** depression, anxiety, stress, factor structure, confirmatory factor analysis, psychometric properties, non-clinical sample, DASS

## Abstract

**Background:** The Depression Anxiety Stress Scales (DASS) are designed to identify quickly and differentiate between the symptoms of depression and anxiety in the non-clinical population. Different versions (original and short) were validated in many cultures. Nevertheless, there are no data of factorial validity of the different versions of this scale in Polish culture. Thus, the aim of this study was to evaluate the factor structure using confirmatory factor analysis (CFA) and internal consistency of DASS-42 (original version) and two short versions (DASS-21 items and DASS-12 items) in the Polish population.

**Methods:** The DASS-42 was administered to a non-clinical sample, broadly representative of the general Polish adult population (*n* = 1,021) in terms of demographic variables. The DASS-21 and DASS-12 version used in this study comprise seven and four items from each of the following corresponding three subscales of the Polish version of DASS-42.

**Results:** There were two models that fitted best for DASS-42: (a) modified three correlated factors (depression, anxiety, and stress) with cross-loadings and (b) second order (general factor of psychological distress) and three factors with cross-loadings. There were also two models that fitted best for DASS-21 and DASS-12: (a) modified three correlated factors (depression, anxiety, and stress) and (b) second order (general factor of psychological distress) and three factors.

**Conclusions:** All three versions of DASS appear to have an acceptable factorial structure. However, the shorter versions (DASS-21 and DASS-12) may be more feasible to use in general medical practice and also be less burdensome to participants.

## Introduction

Data from the World Health Organization (WHO) show that depression and anxiety (pathological level of anxiety) disorders occur worldwide (1). It is well-known for its negative impact on the quality of human life and the social and economic costs (2–4). The results of research under the Global Burden of Disease program conducted from 1990 to 2010 indicated the growing position of depressive disorders among the sources of life burdens for people around the world (5). At the same time, the lack of high quality of epidemiological research is emphasized, which limits the accuracy and usefulness of the obtained results (6). The quality of the research depends, among others, on the research tools used, characterized by satisfactory psychometric properties. Their limited number is the reason for low detection of emotional disorders in the population during routine tests conducted by healthcare professionals (4). One of the most difficult tasks in building a satisfactory tool is the specificity of the symptoms that make up the disorders in question (7). There are difficulties in clinically differentiating between the symptoms of depression and anxiety overlapping. This is despite the formulated concepts explaining the mechanisms of symptom formation (8). The three-factor model of depression and anxiety proposed by Clark and Watson was supposed to remove the above-mentioned inconveniences (9). On its basis, a clinical tool was developed—the Depression Anxiety Stress Scale Long Form questionnaire (DASS-42) (8)—repeatedly empirically verified in terms of psychometric properties (10).

The DASS-42 scale (8) is a self-report tool designed to maximize the differences between symptoms of depression and anxiety and to reveal their common features called stress. This questionnaire has been translated to many languages and has been shown to have a transcultural validity (http://www2.psy.unsw.edu.au/dass/). Currently, it is a widely used tool for screening in non-clinical (11–16) and clinical groups with various diagnoses (10, 17–21). One of the significant limitations of the DASS-42 questionnaire is its length, resulting from the number of items, which slows down the examination time (3, 14). For this reason, among others, shortened versions were created: 21-item (3–20), 18-item (22), 12-item (3), and 9-item (11). The 21-item version is the most used in various clinical and non-clinical groups around the world. It is known that it has quite good psychometric parameters (3, 14, 23). However, the factor structure of the full version of DASS-42 is still not well-defined, which best meets the need to maximize the differences between symptoms of depression and anxiety. In the literature, there are reports from 89 analyzes checking 4, 3, 2, or 1-factor models taking into account and omitting the correlated errors, carried out on data obtained using a different number of items in the full version of the questionnaire (24–26). The search for the DASS factor model with the best psychometric properties is another reason for creating shortened versions of the scale.

The original DASS-42 questionnaire (long version) and their short versions have been translated into many languages and have been empirically evaluated in diverse cultures. Nevertheless, the factorial validity of the different versions of this scale has never been evaluated in the Polish culture. There is only one paper by Zawislak et al. (27) about construct validity and reliability but on the medical student population, not the general population. Given the limitations of the above findings, the aim of this study was to evaluate the factor structure using confirmatory factor analysis (CFA) and internal consistency of three versions of DASS: DASS-42 (23), DASS-21 (14, 26), and DASS-12 (3, 14) in the Polish population. Based on Crawford and Henry (28), we tested the three-factor model of DASS-42 (following the test's original scoring) using CFA. Moreover, we evaluated a three-factor model modified scoring in which cross-loading of specific items was permitted. Based on similar cross-loading items found in exploratory factor analyses (10, 23), Crawford and Henry permitted the anxiety item 9 to also load on the stress factor, the stress item 33 to load on the stress factor, and the anxiety item 30 to load on all three factors. Therefore, the same cross-loadings were permitted in the present sample. Crawford and Henry also tested these various models when the error associated with a particular item was permitted to correlate with the error in another item. This was important because model testing should not only address the relationships between the variables but also between the error terms since the residual of one item may provide information about that associated with another item.

## Methods

### Participants

The non-clinical sample was collected from citizens coming from various towns and villages of southern Poland. The inclusion criteria were age over 18 years, the ability to independently read the text of the questionnaires, and the ability to understand its content and provide answers. No upper age limit has been established. The exclusion criterion was the subjects' current or past psychiatric diagnosis during the completion of the questionnaire. The participants were recruited in person with the help of nursing and medicine students. The participants were people known to the volunteers (family members, friends). Volunteers administered the 42-item full versions of the DASS questionnaire and collected completed questionnaires between January and May 2021. The average time that elapsed from administering the questionnaire to the moment it was completed was 2 weeks. The participants were administered the 42-item full version of the DASS questionnaire. The data of the present study were collected with the help of nursing and medicine students, who volunteered to administer the battery of tests. The volunteers were trained on the distribution, administration, and collection of the questionnaires. After signing an informed consent form, participants were administered a sociodemographic data sheet and the DASS-42 questionnaires. Each respondent was instructed to fill in the questionnaire from research assistants. Participation in this study was anonymous and voluntary. This study was approved by the Ethics Committee of Jagiellonian University (1072.6120.65.2021).

### Materials and Procedure

The DASS-42 measures symptoms of depression, anxiety, and stress (8). It comprises three subscales that each has 14 items: depression (DASS-42 Depression), anxiety (DASS-42 Anxiety), and stress (DASS-42 Stress). Each item is scored on a 4-point Likert scale ranging from 0 (“did not apply to me at all”) to 3 (“applied to me very much”). The scores for the total DASS-42 and for each subscale are summed. In this study, we used the shorter versions of DASS: DASS-21 comprises seven items (DASS-21 Depression; items 3, 10, 17, 26, 31, 38, and 42; DASS-21 Anxiety; items 2, 4, 20, 25, 28, 40, and 41; DASS-21 Stress; items 6, 8, 12, 18, 22, 35, and 39), and DASS-12 (DASS-12 Depression; items 10, 17, 31, and 42; DASS-12 Anxiety; items 20, 28, 40, and 41; DASS-21 Stress; items 6, 22, 35, and 39) comprises four items from each of the following corresponding three subscales of DASS-42, which was suggested by Lee et al. (3), Osman et al. (14), and Henry and Crawford (26). The English version of the DASS-42 was translated into Polish with the permission of the original author (Dr. Lovibond and Dr. Lovibond) in accordance with the translation and back-translation (29). Two bilinguals independently translated the English version to Polish based on semantic equivalence rather than word-to-word equivalence.

### Statistical Analysis

Statistical analysis of the results was done using Statistical Package for Social Sciences (SPSS) version 26 and the Analysis of Moment Structure (AMOS) software version 26. Before conducting the analysis, the data for the 42 items of the DASS were screened for missing values and normality. Missing data were excluded from the analysis. The normality of the distributions was assessed at both the univariate and multivariate levels. Basic information on the variables, i.e., means, standard deviations, standard errors, skewness, and kurtosis, was provided. Internal consistency of the items was measured using Cronbach's alpha coefficient.

Factorial construct validity was done using the three-factor model, and the second-order three-factor model as used in the original DASS study was assessed. We did not analyze the two-factor model because it is rarely supported (3, 30, 31). The validity was assessed using CFA with maximum-likelihood estimation. The sample size of 1,021 in this study satisfied the estimated size requirement (32). For the CFA, multiple fit indices were used. The selected indices were the chi-square statistic (x^2^), the root mean square error of approximation (RMSEA) (33), the standard root mean square residuals (SRMR), the goodness-of-fit index (GFI) (34), and the comparative fit index (CFI) (35). For the RMSEA, values of <0.06, 0.08–0.10, and >0.10 were considered to indicate good, adequate, and poor tests, respectively, and for SRMR, GFI, and CFI, values of <0.05, >0.90, and >0.90 were considered to indicate an acceptable fit, respectively (36, 37). It was suggested that a significant difference in the x^2^ (Δ x^2^) value between a model and its modified model indicates a substantial improvement in model fit (38).

## Results

### Participant's Characteristics

Data were obtained from 1,294 respondents. After removing incomplete and incorrect records (using a listwise deletion technique), the data collected from 1,021 participants (625 female and 396 male, aged 18–83 years old, *M* = 30.67, *SD* = 13.25) were analyzed. The percentage of data missing for each item was in the range between 10.7 and 11.1%. The mean of DASS-42 scores for total scale were 33.08 (*SD* = 22.68); for Depression subscale, 9.22 (*SD* = 8.46); for Anxiety subscale, 8.78 (*SD* = 7.48); and for Stress subscale, 15.08 (*SD* = 9.07). The mean of DASS-21 scores for total scale were 16.61 (*SD* = 11.87); for Depression subscale 5.21 (*SD* = 4.46); for Anxiety subscale, 4.16 (*SD* = 4.14); and for Stress subscale, 7.24 (*SD* = 4.64). The mean of DASS-12 scores for total scale were 9.41 (*SD* = 7.06); for Depression subscale, 3.10 (*SD* = 2.72); for Anxiety subscale, 2.43 (*SD* = 2.67); and for Stress subscale, 3.89 (*SD* = 2.70).

### Base Statistics

As can be seen in Table 1, the distribution of each item showed positive skewness (from 0.11 to 2.63) and mixed (positive and negative) kurtosis (from −1.11 to 6.69). Based on (39), we assumed that skewness between −2 to +2 and kurtosis between −7 to +7 indicated normal distribution of variables.

**Table 1:** Mean scores for three versions of the Depression Anxiety Stress Scale (DASS-42, DASS-21, and DASS-12) and distribution parameters (*n* = 1,021).

**No**	**Item**	***M***	***SD***	**Skewness**	**Kurtosis**
1	I found myself getting upset by quite trivial things.	1.27	0.90	0.34	−0.63
2	I was aware of dryness of my mouth.	0.62	0.83	1.16	0.39
3	I couldn't seem to experience any positive feeling at all.	0.62	0.79	1.14	0.65
4	I experienced breathing difficulty (e.g., excessively rapid breathing, breathlessness in the absence of physical exertion).	0.44	0.76	1.76	2.46
5	I just couldn't seem to get going.	0.30	0.69	2.53	5.88
6	I tended to over-react to situations.	1.24	0.90	0.30	−0.69
7	I had a feeling of shakiness (e.g., legs going to give way).	0.79	0.92	0.91	−0.15
8	I found it difficult to relax.	1.19	0.92	0.40	−0.67
9	I found myself in situations that made me so anxious I was most relieved when they ended.	1.45	1.02	0.11	−1.11
10	I felt that I had nothing to look forward to.	0.70	0.91	1.13	0.26
11	I found myself getting upset rather easily.	1.35	0.95	0.21	−0.86
12	I felt that I was using a lot of nervous energy.	1.13	0.94	0.46	−0.69
13	I felt sad and depressed.	0.99	0.92	0.61	−0.50
14	I found myself getting impatient when I was delayed in any way (e.g., elevators, traffic lights, being kept waiting).	1.24	0.96	0.33	−0.82
15	I had a feeling of faintness.	0.29	0.64	2.43	5.87
16	I felt that I had lost interest in just about everything.	0.65	0.87	1.15	0.36
17	I felt that I wasn't worth much as a person.	0.68	0.87	1.11	0.31
18	I felt I was rather touchy.	1.04	0.94	0.51	−0.70
19	I perspired noticeably (e.g., hands sweaty) in the absence of high temperatures or physical exertion.	0.54	0.83	1.45	1.26
20	I felt scared without any good reason.	0.56	0.79	1.28	0.88
21	I felt that life wasn't worthwhile.	0.39	0.74	1.89	2.80
22	I found it hard to wind down.	0.96	0.89	0.59	−0.49
23	I had difficulty in swallowing.	0.24	0.58	2.63	6.69
24	I couldn't seem to get any enjoyment out of the things I did.	0.64	0.79	1.03	0.33
25	I was aware of the action of my heart in the absence of physical exertion (e.g., sense of heart rate increase,	0.67	0.88	1.18	0.49
	heart missing a beat).				
26	I felt down-hearted and blue.	1.04	0.91	0.54	−0.54
27	I found that I was very irritable.	1.07	0.89	0.45	−0.59
28	I felt I was close to panic.	0.62	0.84	1.17	0.40
29	I found it hard to calm down after something upset me.	0.96	0.93	0.67	−0.48
30	I feared that I would be “thrown” by some trivial but unfamiliar task.	0.77	0.88	0.90	−0.07
31	I was unable to become enthusiastic about anything.	0.54	0.76	1.27	0.82
32	I found it difficult to tolerate interruptions to what I was doing.	0.86	0.88	0.75	−0.28
33	I was in a state of nervous tension.	1.09	0.95	0.50	−0.68
34	I felt I was pretty worthless.	0.51	0.81	1.52	1.48
35	I was intolerant of anything that kept me from getting on with what I was doing.	0.77	0.85	0.94	0.20
36	I felt terrified.	0.53	0.79	1.34	0.88
37	I could see nothing in the future to be hopeful about.	0.52	0.80	1.47	1.35
38	I felt that life was meaningless.	0.46	0.79	1.67	1.89
39	I found myself getting agitated.	0.92	0.89	0.73	−0.24
40	I was worried about situations in which I might panic and make a fool of myself.	0.71	0.90	1.13	0.37
41	I experienced trembling (e.g., in the hands).	0.54	0.83	1.46	1.24
42	I found it difficult to work up the initiative to do things.	1.18	0.99	0.44	−0.84

*M, mean; SD, standard deviation*.

As can be seen in Table 2, the interitem correlation coefficients of the DASS-42 ranged from 0.13 to 0.71, that of DASS-21 ranged from 0.15 to 0.67, and that of DASS-12 ranged from 0.28 to 0.62. All correlations were statistically significant (0.001 < *p* > 0.05). These values indicate that there were no redundant or unrelated items.

**Table 2:** Internal consistency of the three versions of the Depression Anxiety Stress Scale (DASS-42, DASS-21, and DASS-12).

	**DASS-42**		**DASS-21**		**DASS-12**	
	**Interitem correlation**	**Cronbach's α**	**Interitem correlation**	**Cronbach's α**	**Interitem correlation**	**Cronbach's α**
Depression subscale	0.16–0.72	0.93	0.25–0.60	0.86	0.32–0.62	0.77
Anxiety subscale	0.17–0.61	0.89	0.24–0.58	0.84	0.46–0.58	0.81
Stress subscale	0.32–0.71	0.92	0.37–0.67	0.85	0.40–0.47	0.76
Total scale	0.13–0.71	0.96	0.15–0.67	0.93	0.28–0.62	0.89

Cronbach's coefficient was 0.96 for the total DASS-42 and 0.93, 0.89, and 0.92 for the DASS-42 Depression subscale, DASS-42 Anxiety subscale, and DASS-42 stress subscale, respectively. Cronbach's coefficient was 0.93 for the total DASS-21 and 0.86, 0.84, and 0.85 for the DASS-21 Depression subscale, DASS-21 Anxiety subscale, and DASS-21 stress subscale, respectively. Cronbach's coefficient was 0.89 for the total DASS-12 and 0.77, 0.81, and 0.76 for the DASS-12 Depression subscale, DASS-12 Anxiety subscale, and DASS-12 stress subscale, respectively. Both DASS versions, therefore, satisfied internal consistency.

### Factorial Construct Validity

As can be seen in Table 3, three correlated factors of DASS-42 (Model 1) did not represent a good fit of the data (SRMR = 0.056; GFI = 0.797; CFI = 0.845; the only RMSEA = 0.068 have fit adequate criteria). The modified three correlated factors of DASS-42 (Model 1a) represented a significant improvement over Model 1 (Δx^2^ = 1,572.61; *p* <0.001) and yielded a good fit across all indices (RMSEA = 0.053; SRMR = 0.048; CFI = 0.908) except for GFI (0.865). The modified three correlated factors with cross-loadings of DASS-42 (Model 1b) represented a significant improvement over Model 1a (Δ x^2^ = 72.94; *p* <0.001) and yielded a good fit across all indices (RMSEA = 0.052; SRMR = 0.047; CFI = 0.911) except for GFI (0.870), and Model 1c (second-order three factors with cross loadings). For Model 1b and for Model 1c, all the items loaded meaningfully on their designated factors (with a critical ratio value of >1.96), and their standardized factor loading values ranged from 0.345 to 0.797 (except item 9 = 0.122). Details are shown in Supplementary Materials (Supplementary Tables S1A,B; Supplementary Figures S1–S4).

**Table 3:** Model fit indices for the three versions of the Depression Anxiety Stress Scale (DASS-42, DASS-21, and DASS-12).

	**x^**2**^**	**df**	**RMSEA (90% CI)**	**SRMR**	**GFI**	**CFI**	**x^**2**^ difference (Δ x^**2**^)**
DASS-42							Model 1–Model 1a = 1572.61^***^
Model 1: three correlated factors	4611.64^***^	816	0.068 (0.066–0.069)	0.056	0.797	0.845	Model 1a–Model 1b = 72.94^***^
Model 1a: modified three correlated factors	3039.03^***^	792	0.053 (0.051–0.055)	0.048	0.865	0.908	
Model 1b: modified three correlated factors with cross loadings	2966.09^***^	788	0.052 (0.050–0.054)	0.047	0.870	0.911	
Model 1c: second-order three factors with cross loadings	2966.09^***^	788	0.052 (0.050–0.054)	0.047	0.870	0.911	
DASS-21							Model 2–Model 2a = 389.11^***^
Model 2: three correlated factors	1103.63^***^	186	0.070 (0.066–0.074)	0.046	0.901	0.908	
Model 2a: modified three correlated factors	714.52^***^	176	0.055 (0.051–0.061)	0.035	0.936	0.946	
Model 2b: second-order three factors	714.52^***^	176	0.055 (0.051–0.059)	0.035	0.936	0.946	
DASS-12							Model 3–Model 3a = 83.15^***^
Model 3: three correlated factors	236.19^***^	51	0.060 (0.052–0.067)	0.037	0.964	0.961	
Model 3a: modified three correlated factors	153.04^***^	49	0.046 (0.038–0.054)	0.026	0.976	0.978	
Model 3b: second-order three factors	153.04^***^	49	0.046 (0.038–0.054)	0.026	0.976	0.978	

*** *p < 0.001*.

Three correlated factors of DASS-21 (Model 2) represented a good fit of the data (RMSEA = 0.070; SRMR = 0.046; GFI = 0.901; CFI = 0.908). The modified three correlated factors of DASS-21 (Model 2a) represented a significant improvement over Model 2 (Δ x^2^ = 389.11; *p* <0.001) and yielded a good fit across all indices (RMSEA = 0.055; SRMR = 0.035; GFI = 0.936; CFI = 0.946), and Model 2b (second-order three factors). For Model 2a and for Model 2b, all the items loaded meaningfully on their designated factors (with a critical ratio value of >1.96), and their standardized factor loading values ranged from 0.408 to 0.776. Details are shown in Supplementary Materials (Supplementary Tables S2A,B; Supplementary Figures S5–S7).

Three correlated factors of DASS-12 (Model 3) represented a good fit of the data (RMSEA = 0.060; SRMR = 0.037; GFI = 0.964; CFI = 0.961). The modified three correlated factors of DASS-12 (Model 3a) represented a significant improvement over Model 3 (Δ x^2^ = 83.15; *p* <0.001) and yielded a good fit across all indices (RMSEA = 0.046; SRMR = 0.026; GFI = 0.976; CFI = 0.978), and Model 3b (second-order three factors). For Model 3a and for Model 3b, all the items loaded meaningfully on their designated factors (with a critical ratio value of >1.96), and their standardized factor loading values ranged from 0.529 to 0.767. Details are shown in Supplementary Materials (Supplementary Tables S3A,B; Supplementary Figures S8–S10).

## Discussion

The study is the first analysis of the DASS factor structure using the CFA method in the general population of adult Poles. It is also the first study to verify the factor structure of three versions of the tool, namely, full (Long Form) and two shortened, i.e., DASS-42, DASS-21 and DASS-12.

Differences in the fit of the models corresponding to the three versions of the questionnaire were obtained. Model-1 with three correlated factors DASS-42 did not get a good fit. For this reason, a modified model with three correlated factors was checked, taking into account intercorrelations (relationships) between measurement errors of individual items. This one turned out to be much better than the previous one and showed a good fit on all metrics except GFI. In the next stage of the analysis, the model of modified three correlated factors with cross-loadings was checked. Item 9, an anxiety factor charger, was allowed to load the stress factor as well; item 33, a stress factor charger, was allowed to load the anxiety factor as well. Item 30 was also allowed to load all three factors. This model proved to be better than the previous model and showed better fit in all indicators except GFI. Finally, the second-order three factors model with cross-loadings was tested. The second-order factor was called “general negative emotion” (8) or “general psychological distress” (26). This model obtained similar indicators as the previous one.

For DASS-21, model-2 with three correlated factors showed a good fit. The procedure used in the long form of questionnaire (DASS-42) analysis was repeated, checking the modified three correlated factors model and the second-order three factors with cross-loading model. Both models mentioned revealed the same best match rates.

In the case of the model-3 (DASS-12), which proved to be a good fit in the version with three correlated factors, the applied procedure resulted in an improvement of the indicators. In addition, in this model, the version with three modified correlated factors and the second-order factor obtained similar, best fit rates.

The standardized loading factor for each of the three models ranged from 0.345 to 0.797, only in the case of DASS-42 one item (9)—“I found myself in situations that made me so anxious. I was most relieved when they ended”—loaded definitely low. In the studies by Clara et al. (10) and Antony et al. (23), there was also a problem with the above-mentioned item, which was loaded with both the stress and anxiety factors. Brown et al. (40) suggested that this item should belong to both factors at the same time, creating the so-called modified three-factor model. However, a study by Clara et al. (10), conducted in the clinical population, showed that the use of a model in which item 9 cross-loaded two factors simultaneously only slightly improved the psychometric properties of the basic Lovibond and Lovibond model (8). According to the authors, it is better to stop at the classic three-factor model.

The results obtained in the study were consistent with the results presented by other researchers. As for the DASS-42, the own study of the Persian version of the tool (13), also conducted in the non-clinical group, indicated the model with three correlated factors as having the best fit. However, unlike our research, the above-mentioned results resulted in the removal of four items from the full version of the scale. The exploratory analysis carried out by Antony et al. (23) also confirmed the structure of the tool made up of three correlated factors. In the case of DASS-21, both in the Clara et al. (10) study and in ours, we observed better fitting parameters compared to the full version. Sharma et al. (41), while examining the structure of the Indian version of DASS-21, observed high parameters for the model with three correlated factors using confirmatory analysis. They were slightly higher than those obtained in our research. In the study by Henry and Crawford (26), an optimal fit was observed for the bifactor (quadri-partite structure) model consisting of the general anxiety factor and three specific factors—orthogonal (depression, anxiety, stress). The structure of this model was similar to our structure of the second-order three factors model (-2b model). The research of Lee et al. on the Korean versions of DASS-21 and DASS-12 (3) using confirmatory analysis confirmed a good fit of both the three-factor model and the three-factor model with the second-order factor. The authors checked five models for each version of the scale, starting from the one-factor model, which revealed a poor fit, through the basic three-factor model, which also had poor parameters. The three-factor model modified from two covariance error terms with much better parameters and ending with the second-order tree-factor model with modification of two covariance error terms. This model contained a second level of a factor named after Lovibond and Lovibond (8) “general negative emotion,” similar to the study by Henry and Crawford (26). It was as good as the three-factor but explained more of the variance in the results and was considered better than the alternative models. Studies by Osman et al. (14) indicated the best fit of two models: second-order factor and bifactor with the general factor G. In the case of DASS-12, modified three-factors model with two covariance error terms turned out to be the best match. The results obtained by us were definitely better than those reported by Osman et al. (14), who reduced the DASS 21 by selecting 12 items for the set.

As in the case of the research carried out with the use of other cultural versions of the questionnaire, it was observed that three-factors crossing models and models with a second-order factor were better suited than the models taking into account only the intercorrelation of items. The DASS-42, DASS-21, and DASS-12 questionnaires have equivalent psychometric parameters and can be successfully used in clinical screening and research studies. In the Polish version, they differentiate well the three mental states, as intended by the authors of the original versions. The preliminary analysis showed that all three versions of the questionnaire (DASS-42, DASS-21, and DASS-12) are characterized by good internal consistency (good to excellent) for both whole scales and subscales, ranging between 0.76 and 0.96. The items making up each of the subscales and the overall score were not redundant and unrelated. Similar results were obtained in studies of other language versions (3). The lower Cronbach's α values obtained in our study of the DASS-12 version may result from the lower number of items and were similar to the values in the Korean studies (3).

In conclusion, DASS-42, DASS-21, and DASS-12 well-differentiate the features of depression, anxiety, and stress. As noted by Antony et al. (23), DASS-21 differentiates clinical groups in a manner comparable to DASS-42. Guided by the results of the DASS-12 research, for practical reasons, it is suggested to use the shortest version of the scale, which has psychometric properties comparable to the full version and at the same time significantly reduces the duration of the study and is less burdensome to use for the respondents. During the current coronavirus disease 2019 (COVID-19) pandemic, DASS tool allows GPs or occupational health practitioners to quickly identify patients for the presence of symptoms of depression, anxiety, and stress. It can also be used before consulting general practitioners and occupational medicine physicians. The results obtained in the screening tests, once in the hands of doctors, can be used in the diagnostic process. As Nieuwenhuijsen et al. (4) noted, routine dissemination of DASS to employees can help occupational health professionals in the early diagnostic process. DASS can also help clinicians identify patients at high risk of comorbid depressive disorders (42). DASS can also be used to study the dimensions of human mental functioning and to measure a controlled variable in comparative studies of different clinical groups, e.g., in the field of cognitive functioning.

Contrary to the approach used in another study of the Polish version of the scale (27), we tried to find a model that would be more consistent with the theory underlying the questionnaire than with the analysis of its items. Moreover, Zawislak et al. (27) conducted exploratory factor analysis (EFA) of DASS-21 on Polish medical students, not the general population. Statistical analysis identified four factors of DASS-21. Because factor 4 consisted of only three items, the authors decided to conduct EFA in a modified version renamed DASS-18. Results of this study showed limited data on the factor structure of DASS to compare with our study. We wanted to broaden the knowledge of the original form of the questionnaire, matching the versions with different numbers of items [original and two shorter versions, verified in previous studies of different populations (3, 10–14, 16–20, 26)] rather than to check its structure typical of Polish conditions.

The strength of the study was to check three versions of the DASS, full and two abbreviated. In our study, all three versions obtained good psychometric properties. Looking for a solution with the best possible adaptation, we tested various first- and second-order models, without changing the number of items in each of the DASS versions. One of the limitations of our study is to limit itself to the assessment of the tool's factor structure (no assessment of external validity and stability over time). However, we tried to go beyond this limitation by checking the internal consistency and intercorrelations that achieved satisfactory values. Another limitation of our study is the lack of comparison to clinical samples. Thus, we planned to conduct research to explore diagnostic validity based on the comparisons of the results of healthy people and people with various forms of depressive and anxiety disorders. Moreover, we have further validation studies ahead of us: the assessment of congruent validity and the retest reliability test in the longitudinal study. The third limitation was that the choice of the sample was non-random; however, participants came from various towns and provinces of the country.

It should be remembered that DASS is a questionnaire used only for screening; it does not allow to distinguish different forms of depression, e.g., severe depression, depressive disorders in the course of somatic diseases, adaptation disorders, and various forms of anxiety disorders. Nevertheless, our results suggest that three Polish versions of DASS (DASS-42, DASS-21, and DASS-12) are a valuable tool that allows for diagnostic and scientific research in the field of health psychology, clinical psychology, and psychiatry. Moreover, short versions may be more feasible to use in a busy practice and also be less burdensome to respondents.

## Conclusions

The Depression Anxiety Stress Scales (DASS) are designed to quickly identify and differentiate between the symptoms of depression and anxiety in the non-clinical population, and different versions (full and short) were validated in many cultures. Nevertheless, there are no data of factorial validity of the different versions of this scale in the Polish culture. Thus, the aim of this study was to evaluate the factor structure using confirmatory factor analysis (CFA) of DASS-42 (full version) and two short versions (DASS-21 items and DASS-12 items) in the Polish population. Our results suggest that all three versions of DASS appear to have an acceptable factorial structure. However, the shorter versions (DASS-21 and DASS-12) may be more feasible to use in general medical practice and also be less burdensome to participants.

## Data Availability Statement

The raw data supporting the conclusions of this article will be made available by the authors, without undue reservation.

## Ethics Statement

The studies involving human participants were reviewed and approved by Ethics Committee of Jagiellonian University (1072.6120.65.2021). The patients/participants provided their written informed consent to participate in this study.

## Author Contributions

MM-S was the principal coordinator of the research project, was involved in the study design, took part in patient recruitment, managed literature searches and analyses, performed statistical analysis, and wrote the first draft of the manuscript. ET was involved in the study design, managed literature searches and analyses, performed statistical analysis, and wrote the first draft of the manuscript. MZ was involved in the study design, took part in patient recruitment, managed literature searches and analyses, performed statistical analysis, and wrote the first draft of the manuscript. KA, JM, and AM were involved in the study design and corrected the manuscript. All authors contributed to and have approved the final manuscript.

## Conflict of Interest

The authors declare that the research was conducted in the absence of any commercial or financial relationships that could be construed as a potential conflict of interest. The reviewer PL declared a shared affiliation, with no collaboration, with one of the authors ET to the handling editor at the time of the review.

## Publisher's Note

All claims expressed in this article are solely those of the authors and do not necessarily represent those of their affiliated organizations, or those of the publisher, the editors and the reviewers. Any product that may be evaluated in this article, or claim that may be made by its manufacturer, is not guaranteed or endorsed by the publisher.
