# Supplementary material for: Confirmatory Factor Analysis of Three Versions of the Depression Anxiety Stress Scale (DASS-42, DASS-21, and DASS-12) in Polish Adults
Source: Front Psychiatry. 2022 Jan 4;12:770532. doi: 10.3389/fpsyt.2021.770532 (PMC8764392; doi:10.3389/fpsyt.2021.770532)
Supplement: Supplementary file 1 [file Table_1.DOCX]

**TABLE 1A SUPPLEMENTARY** Standardized regression weights for Model 1c and for Model 1b of the Depression Anxiety Stress Scale (DASS-42).

| Depression subscale | <--- | General stress | 0.888 |
| --- | --- | --- | --- |
| Stress subscale | <--- | General stress | 0.870 |
| Anxiety subscale | <--- | General stress | 0.923 |
| DASS37 | <--- | Depression subscale | 0.750 |
| DASS38 | <--- | Depression subscale | 0.724 |
| DASS10 | <--- | Depression subscale | 0.721 |
| DASS34 | <--- | Depression subscale | 0.757 |
| DASS21 | <--- | Depression subscale | 0.666 |
| DASS31 | <--- | Depression subscale | 0.797 |
| DASS17 | <--- | Depression subscale | 0.734 |
| DASS16 | <--- | Depression subscale | 0.789 |
| DASS3 | <--- | Depression subscale | 0.682 |
| DASS26 | <--- | Depression subscale | 0.736 |
| DASS24 | <--- | Depression subscale | 0.772 |
| DASS13 | <--- | Depression subscale | 0.743 |
| DASS42 | <--- | Depression subscale | 0.499 |
| DASS5 | <--- | Depression subscale | 0.388 |
| DASS41 | <--- | Anxiety subscale | 0.692 |
| DASS7 | <--- | Anxiety subscale | 0.629 |
| DASS15 | <--- | Anxiety subscale | 0.566 |
| DASS4 | <--- | Anxiety subscale | 0.597 |
| DASS25 | <--- | Anxiety subscale | 0.675 |
| DASS28 | <--- | Anxiety subscale | 0.745 |
| DASS23 | <--- | Anxiety subscale | 0.533 |
| DASS20 | <--- | Anxiety subscale | 0.739 |
| DASS36 | <--- | Anxiety subscale | 0.695 |
| DASS40 | <--- | Anxiety subscale | 0.667 |
| DASS2 | <--- | Anxiety subscale | 0.426 |
| DASS9 | <--- | Anxiety subscale | 0.122 |
| DASS19 | <--- | Anxiety subscale | 0.506 |
| DASS30 | <--- | Anxiety subscale | 0.345 |
| DASS1 | <--- | Stress subscale | 0.628 |
| DASS11 | <--- | Stress subscale | 0.698 |
| DASS27 | <--- | Stress subscale | 0.759 |
| DASS39 | <--- | Stress subscale | 0.675 |
| DASS18 | <--- | Stress subscale | 0.630 |
| DASS35 | <--- | Stress subscale | 0.625 |
| DASS6 | <--- | Stress subscale | 0.700 |
| DASS14 | <--- | Stress subscale | 0.534 |
| DASS8 | <--- | Stress subscale | 0.680 |
| DASS29 | <--- | Stress subscale | 0.711 |
| DASS32 | <--- | Stress subscale | 0.630 |
| DASS12 | <--- | Stress subscale | 0.700 |
| DASS22 | <--- | Stress subscale | 0.678 |
| DASS33 | <--- | Stress subscale | 0.686 |
| DASS33 | <--- | Anxiety subscale | 0.045 |
| DASS30 | <--- | Stress subscale | 0.206 |
| DASS30 | <--- | Depression subscale | 0.158 |
| DASS9 | <--- | Stress subscale | 0.401 |

**TABLE 1B SUPPLEMENTARY** Squared multiple correlations for Model 1c and for Model 1b of the Depression Anxiety Stress Scale (DASS-42).

| Stress subscale | 0.757 |
| --- | --- |
| Anxiety subscale | 0.853 |
| Depression subscale | 0.789 |
| DASS33 | 0.522 |
| DASS22 | 0.459 |
| DASS12 | 0.490 |
| DASS32 | 0.397 |
| DASS29 | 0.505 |
| DASS8 | 0.462 |
| DASS14 | 0.285 |
| DASS6 | 0.490 |
| DASS35 | 0.390 |
| DASS18 | 0.396 |
| DASS39 | 0.456 |
| DASS27 | 0.577 |
| DASS11 | 0.487 |
| DASS1 | 0.394 |
| DASS30 | 0.441 |
| DASS19 | 0.256 |
| DASS9 | 0.254 |
| DASS2 | 0.181 |
| DASS40 | 0.445 |
| DASS36 | 0.483 |
| DASS20 | 0.547 |
| DASS23 | 0.284 |
| DASS28 | 0.554 |
| DASS25 | 0.456 |
| DASS4 | 0.357 |
| DASS15 | 0.321 |
| DASS7 | 0.395 |
| DASS41 | 0.480 |
| DASS5 | 0.150 |
| DASS42 | 0.249 |
| DASS13 | 0.552 |
| DASS24 | 0.596 |
| DASS26 | 0.542 |
| DASS3 | 0.465 |
| DASS16 | 0.623 |
| DASS17 | 0.539 |
| DASS31 | 0.634 |
| DASS21 | 0.444 |
| DASS34 | 0.574 |
| DASS10 | 0.520 |
| DASS38 | 0.524 |
| DASS37 | 0.563 |
