# Supplementary material for: Confirmatory Factor Analysis of Three Versions of the Depression Anxiety Stress Scale (DASS-42, DASS-21, and DASS-12) in Polish Adults
Source: Front Psychiatry. 2022 Jan 4;12:770532. doi: 10.3389/fpsyt.2021.770532 (PMC8764392; doi:10.3389/fpsyt.2021.770532)
Supplement: Supplementary file 2 [file Table_2.DOCX]

**TABLE 2A SUPPLEMENTARY** Standardized regression weights for Model 2b of the Depression Anxiety Stress Scale (DASS-21).

| Anxiety subscale | <--- | General stress | 0.901 |
| --- | --- | --- | --- |
| Depression subscale | <--- | General stress | 0.934 |
| Stress subscale | <--- | General stress | 0.948 |
| DASS38 | <--- | Depression subscale | 0.653 |
| DASS10 | <--- | Depression subscale | 0.646 |
| DASS3 | <--- | Depression subscale | 0.687 |
| DASS31 | <--- | Depression subscale | 0.776 |
| DASS17 | <--- | Depression subscale | 0.686 |
| DASS26 | <--- | Depression subscale | 0.771 |
| DASS42 | <--- | Depression subscale | 0.524 |
| DASS25 | <--- | Anxiety subscale | 0.683 |
| DASS4 | <--- | Anxiety subscale | 0.578 |
| DASS41 | <--- | Anxiety subscale | 0.680 |
| DASS28 | <--- | Anxiety subscale | 0.758 |
| DASS20 | <--- | Anxiety subscale | 0.732 |
| DASS40 | <--- | Anxiety subscale | 0.687 |
| DASS2 | <--- | Anxiety subscale | 0.408 |
| DASS35 | <--- | Stress subscale | 0.585 |
| DASS18 | <--- | Stress subscale | 0.635 |
| DASS8 | <--- | Stress subscale | 0.694 |
| DASS39 | <--- | Stress subscale | 0.699 |
| DASS12 | <--- | Stress subscale | 0.639 |
| DASS22 | <--- | Stress subscale | 0.699 |
| DASS6 | <--- | Stress subscale | 0.643 |

**TABLE 2B SUPPLEMENTARY** Squared multiple correlations for Model 2b of the Depression Anxiety Stress Scale (DASS-21).

| Stress subscale | 0.899 |
| --- | --- |
| Anxiety subscale | 0.812 |
| Depression subscale | 0.872 |
| DASS6 | 0.413 |
| DASS22 | 0.489 |
| DASS12 | 0.408 |
| DASS39 | 0.488 |
| DASS8 | 0.481 |
| DASS18 | 0.403 |
| DASS35 | 0.342 |
| DASS2 | 0.166 |
| DASS40 | 0.472 |
| DASS20 | 0.536 |
| DASS28 | 0.575 |
| DASS41 | 0.463 |
| DASS4 | 0.334 |
| DASS25 | 0.466 |
| DASS42 | 0.275 |
| DASS26 | 0.595 |
| DASS17 | 0.471 |
| DASS31 | 0.603 |
| DASS3 | 0.472 |
| DASS10 | 0.418 |
| DASS38 | 0.426 |
