# Supplementary material for: Confirmatory Factor Analysis of Three Versions of the Depression Anxiety Stress Scale (DASS-42, DASS-21, and DASS-12) in Polish Adults
Source: Front Psychiatry. 2022 Jan 4;12:770532. doi: 10.3389/fpsyt.2021.770532 (PMC8764392; doi:10.3389/fpsyt.2021.770532)
Supplement: Supplementary file 3 [file Table_3.DOCX]

**TABLE 3A SUPPLEMENTARY** Standardized regression weights for Model 3b of the Depression Anxiety Stress Scale (DASS-12).

| Depression subscale | <--- | General stress | 0.942 |
| --- | --- | --- | --- |
| Anxiety subscale | <--- | General stress | 0.920 |
| Stress subscale | <--- | General stress | 0.911 |
| DASS41 | <--- | Anxiety subscale | 0.656 |
| DASS40 | <--- | Anxiety subscale | 0.716 |
| DASS28 | <--- | Anxiety subscale | 0.767 |
| DASS20 | <--- | Anxiety subscale | 0.734 |
| DASS22 | <--- | Stress subscale | 0.711 |
| DASS6 | <--- | Stress subscale | 0.636 |
| DASS39 | <--- | Stress subscale | 0.696 |
| DASS35 | <--- | Stress subscale | 0.601 |
| DASS42 | <--- | Depression subscale | 0.529 |
| DASS10 | <--- | Depression subscale | 0.604 |
| DASS31 | <--- | Depression subscale | 0.758 |
| DASS17 | <--- | Depression subscale | 0.691 |

**TABLE 3B SUPPLEMENTARY** Squared multiple correlations for Model 3b of the Depression Anxiety Stress Scale (DASS-12).

| Depression subscale | 0.888 |
| --- | --- |
| Stress subscale | 0.829 |
| Anxiety subscale | 0.847 |
| DASS35 | 0.361 |
| DASS39 | 0.485 |
| DASS6 | 0.404 |
| DASS22 | 0.505 |
| DASS20 | 0.538 |
| DASS28 | 0.588 |
| DASS40 | 0.513 |
| DASS41 | 0.430 |
| DASS17 | 0.477 |
| DASS31 | 0.574 |
| DASS10 | 0.365 |
| DASS42 | 0.280 |
