# Supplementary figures and images for: Confirmatory Factor Analysis of Three Versions of the Depression Anxiety Stress Scale (DASS-42, DASS-21, and DASS-12) in Polish Adults
Source: Front Psychiatry. 2022 Jan 4;12:770532. doi: 10.3389/fpsyt.2021.770532 (PMC8764392; doi:10.3389/fpsyt.2021.770532)

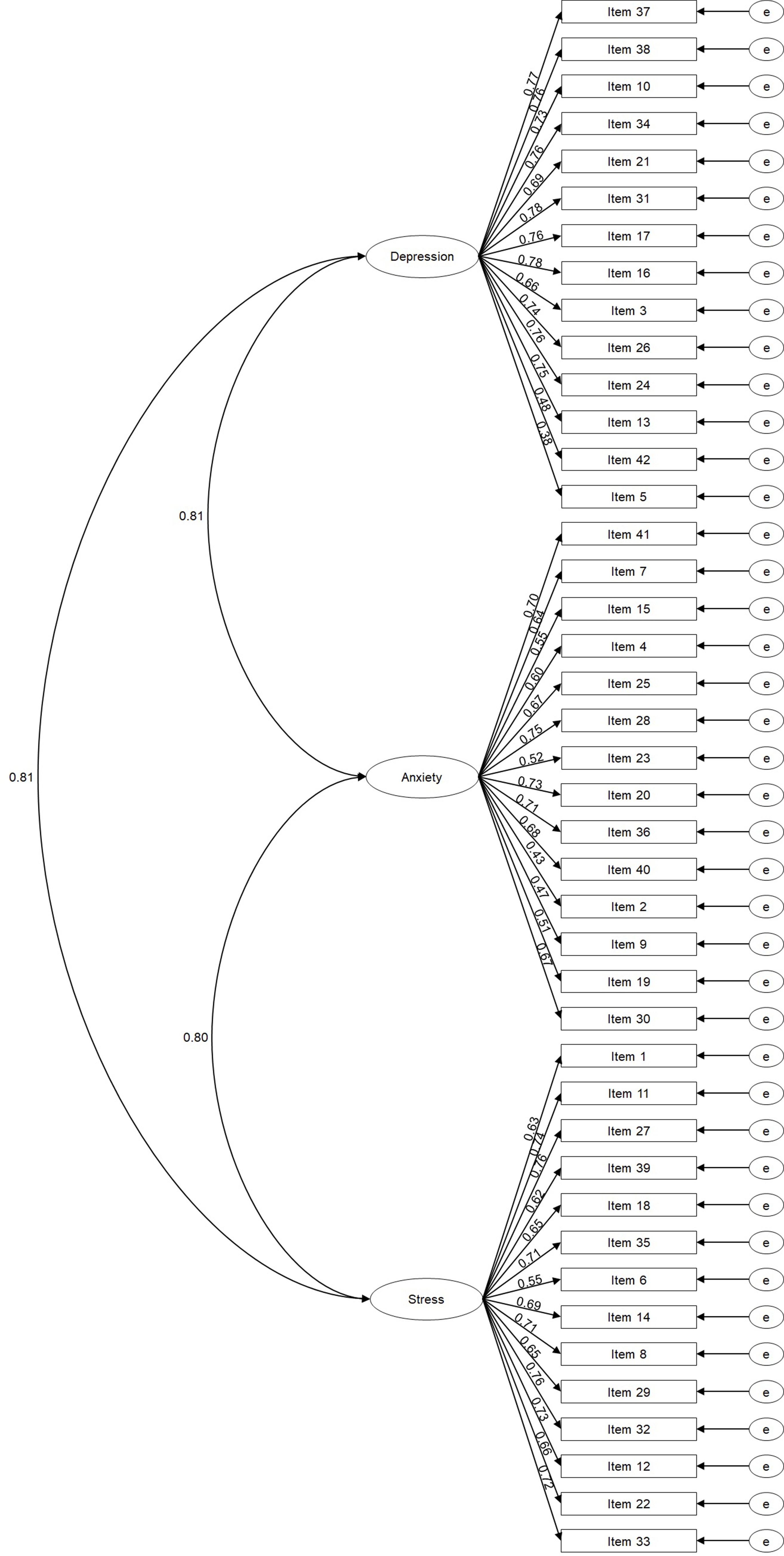

Supplement: Supplementary file 4 [file Image_1.JPG]

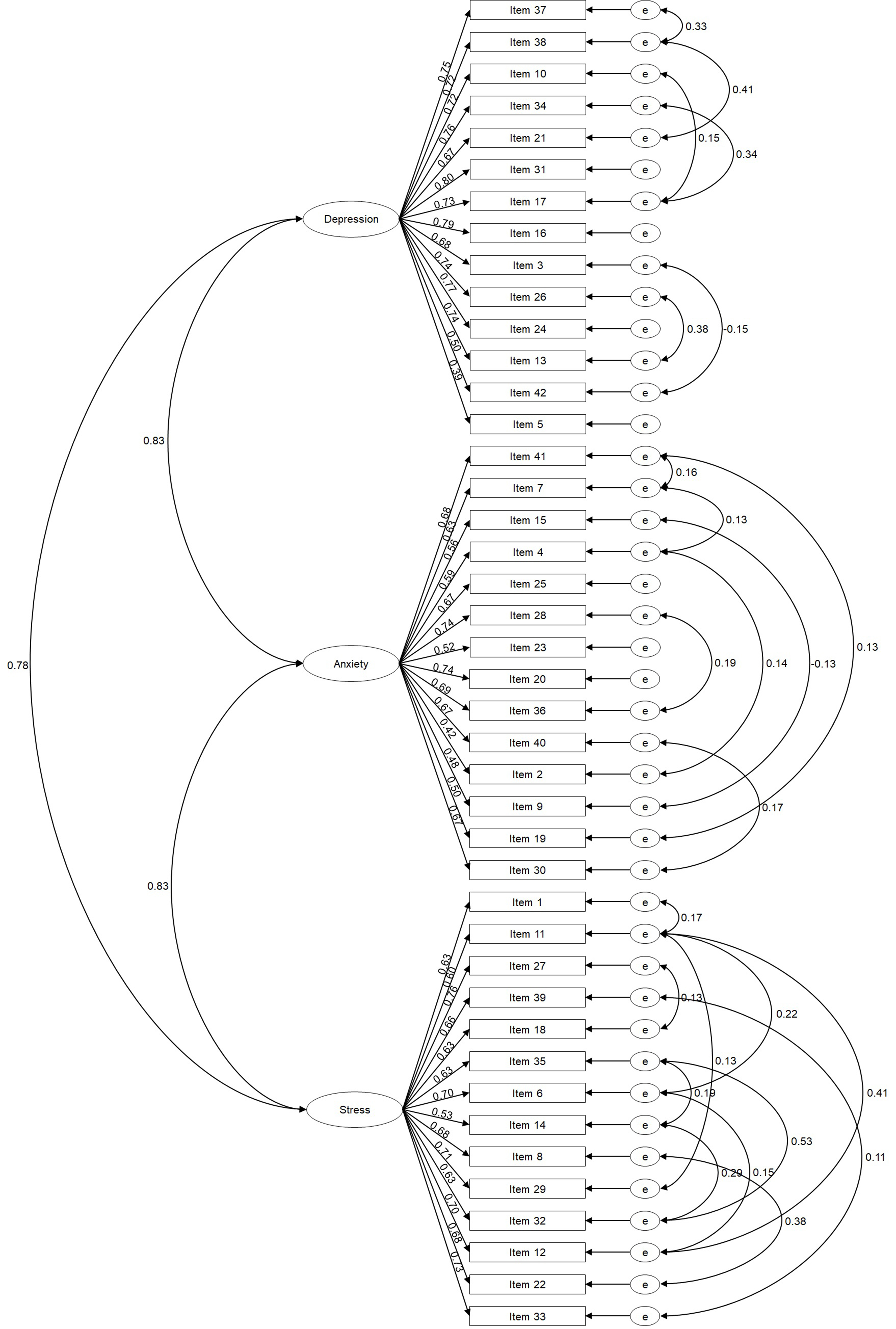

Supplement: Supplementary file 5 [file Image_2.JPG]

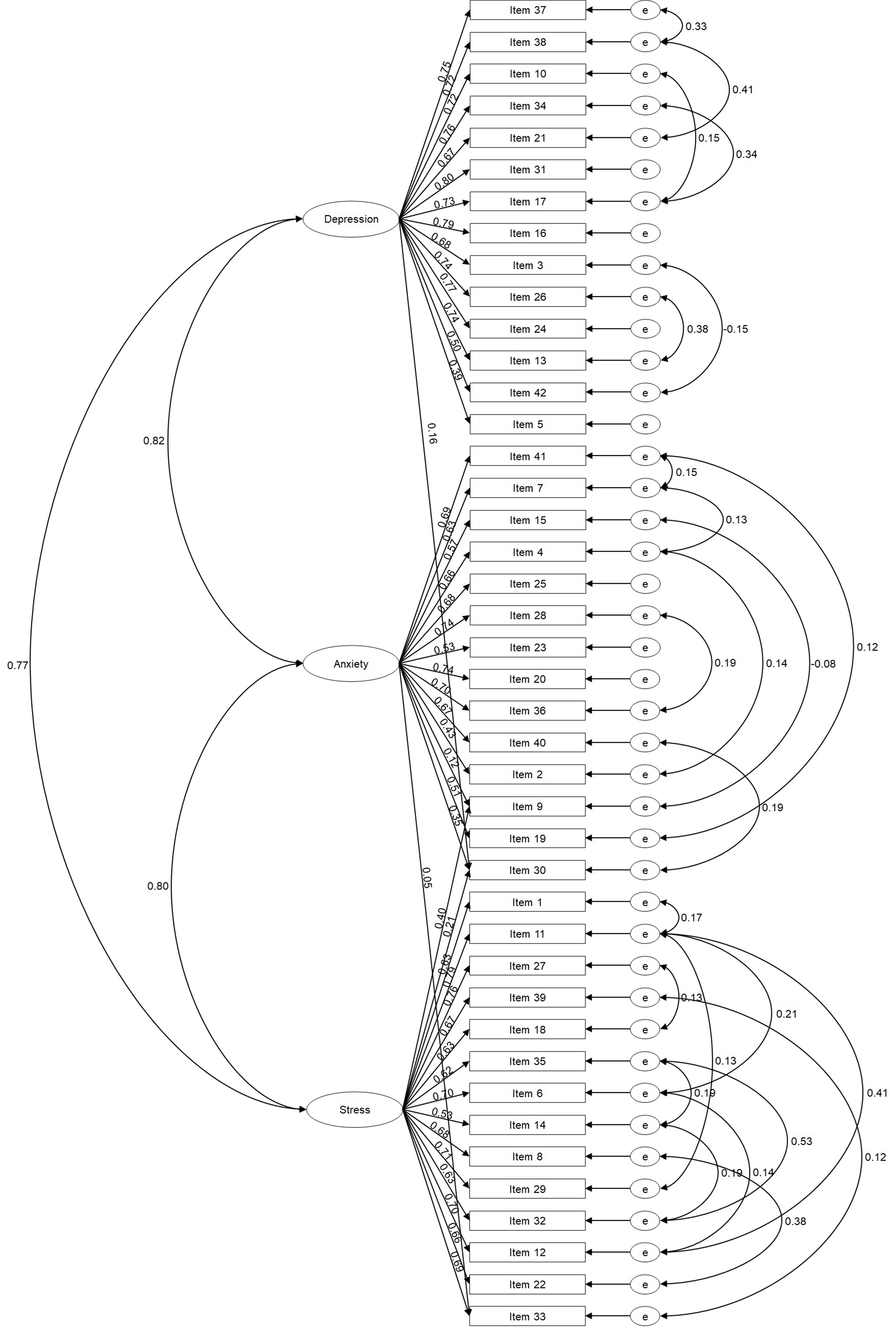

Supplement: Supplementary file 6 [file Image_3.JPG]

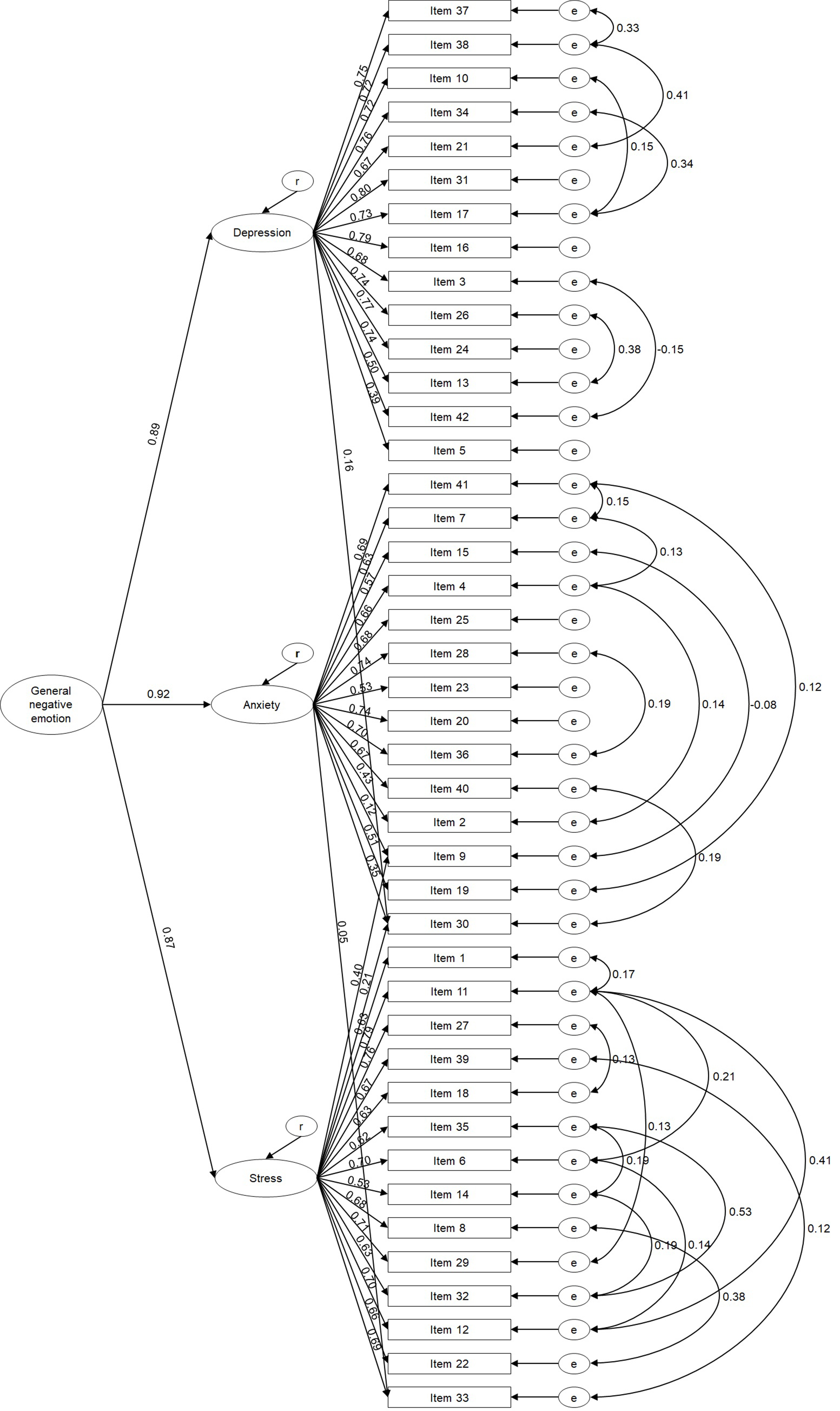

Supplement: Supplementary file 7 [file Image_4.JPG]

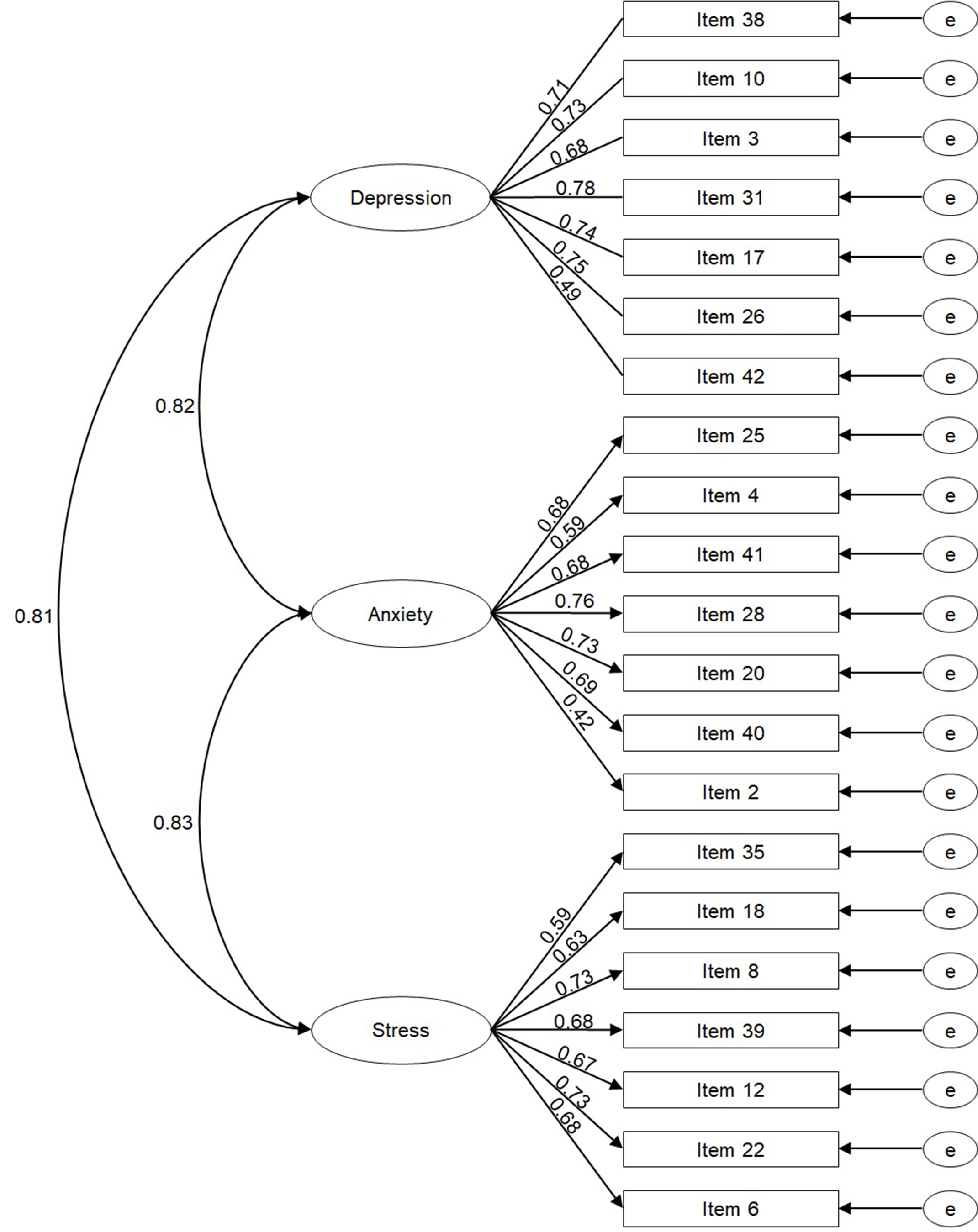

Supplement: Supplementary file 8 [file Image_5.JPG]

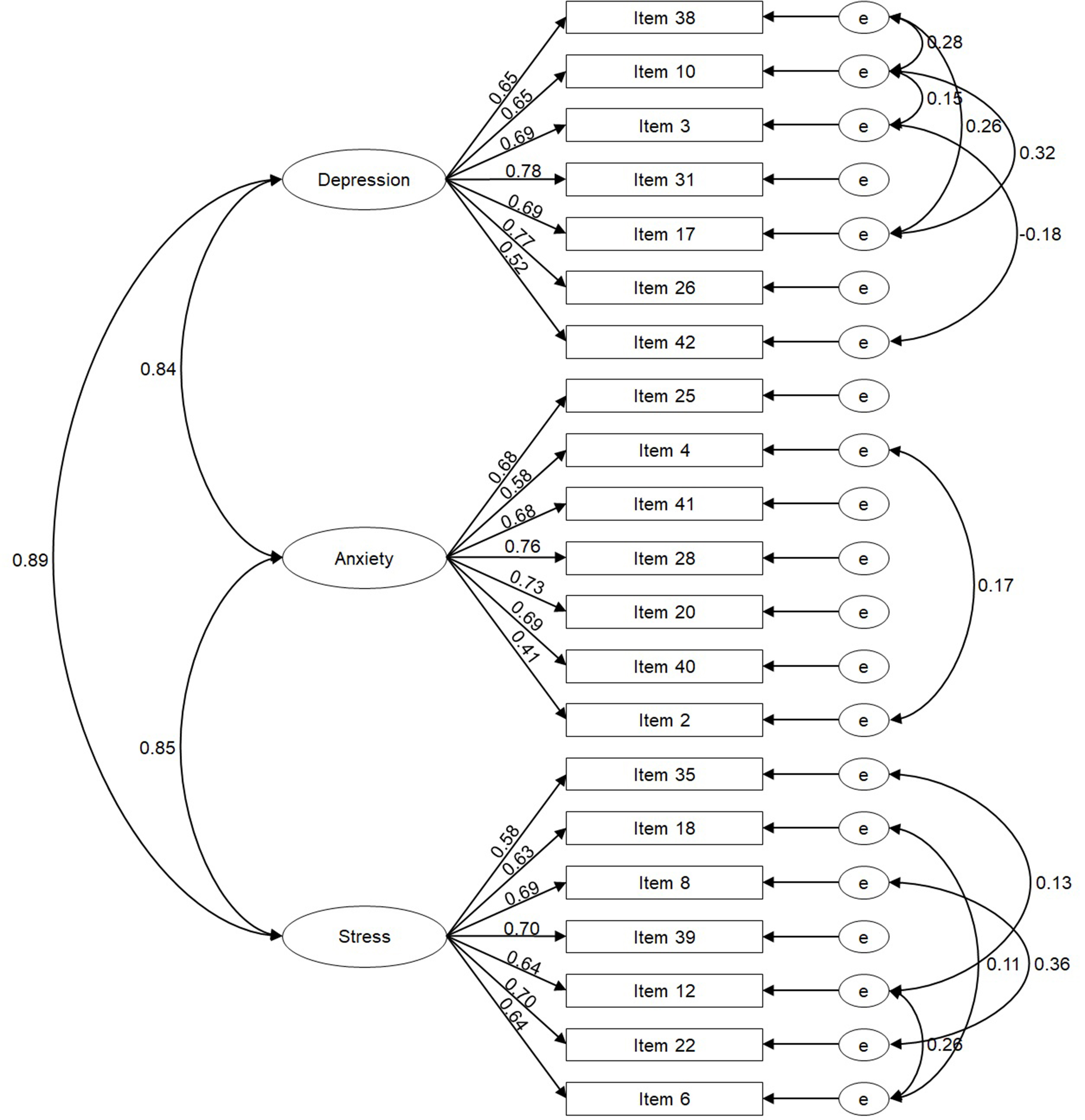

Supplement: Supplementary file 9 [file Image_6.JPG]

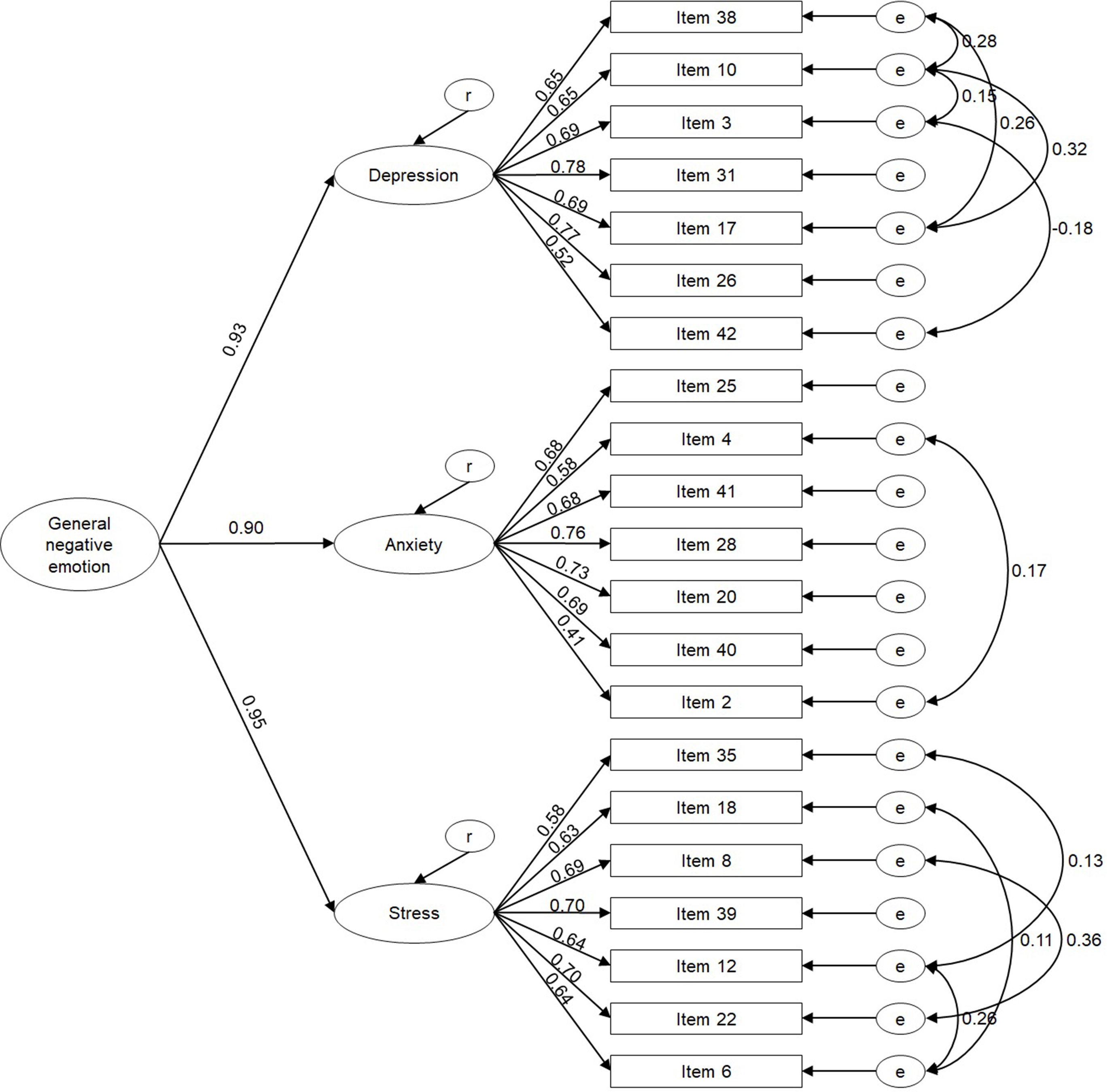

Supplement: Supplementary file 10 [file Image_7.JPG]

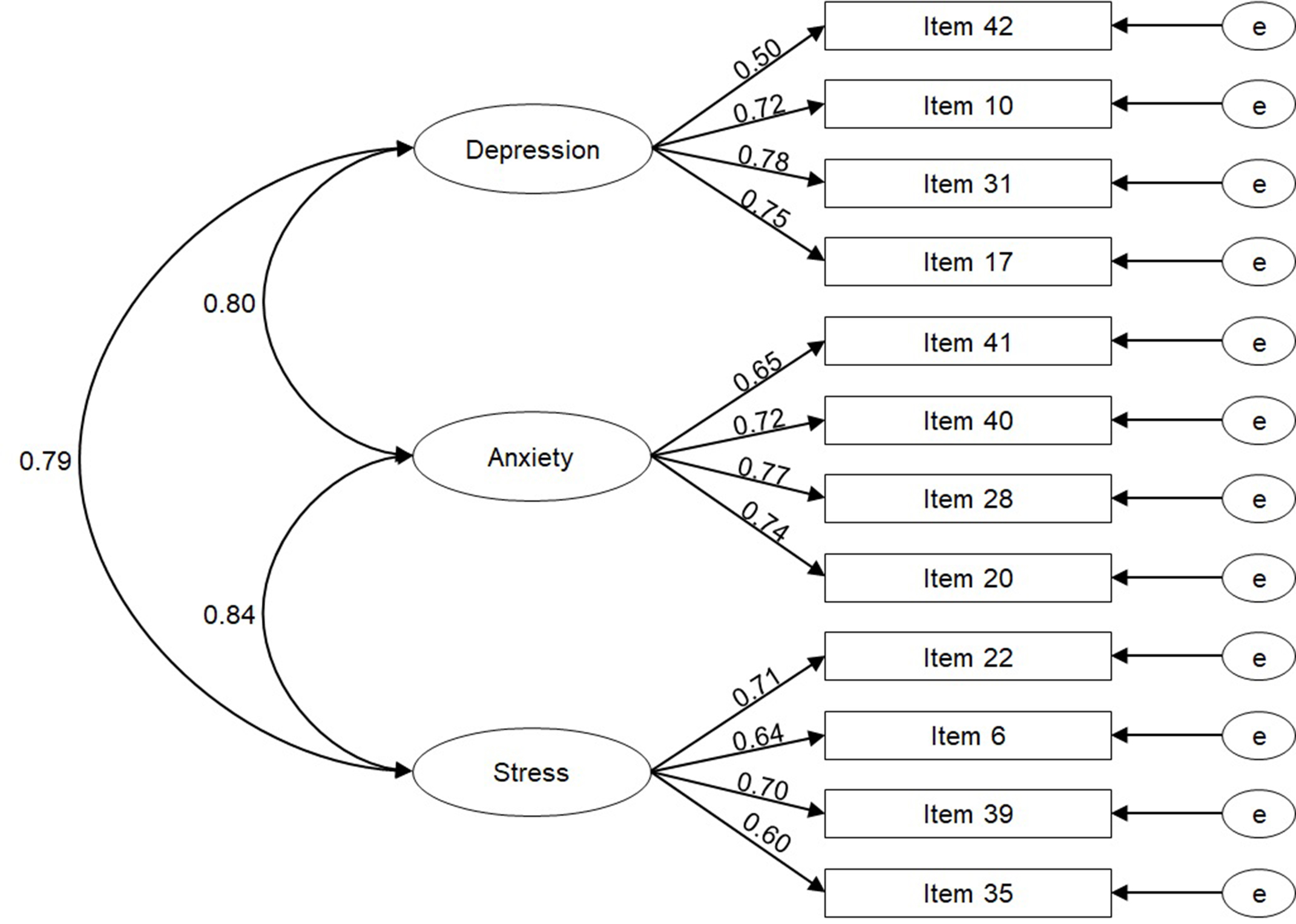

Supplement: Supplementary file 11 [file Image_8.JPG]

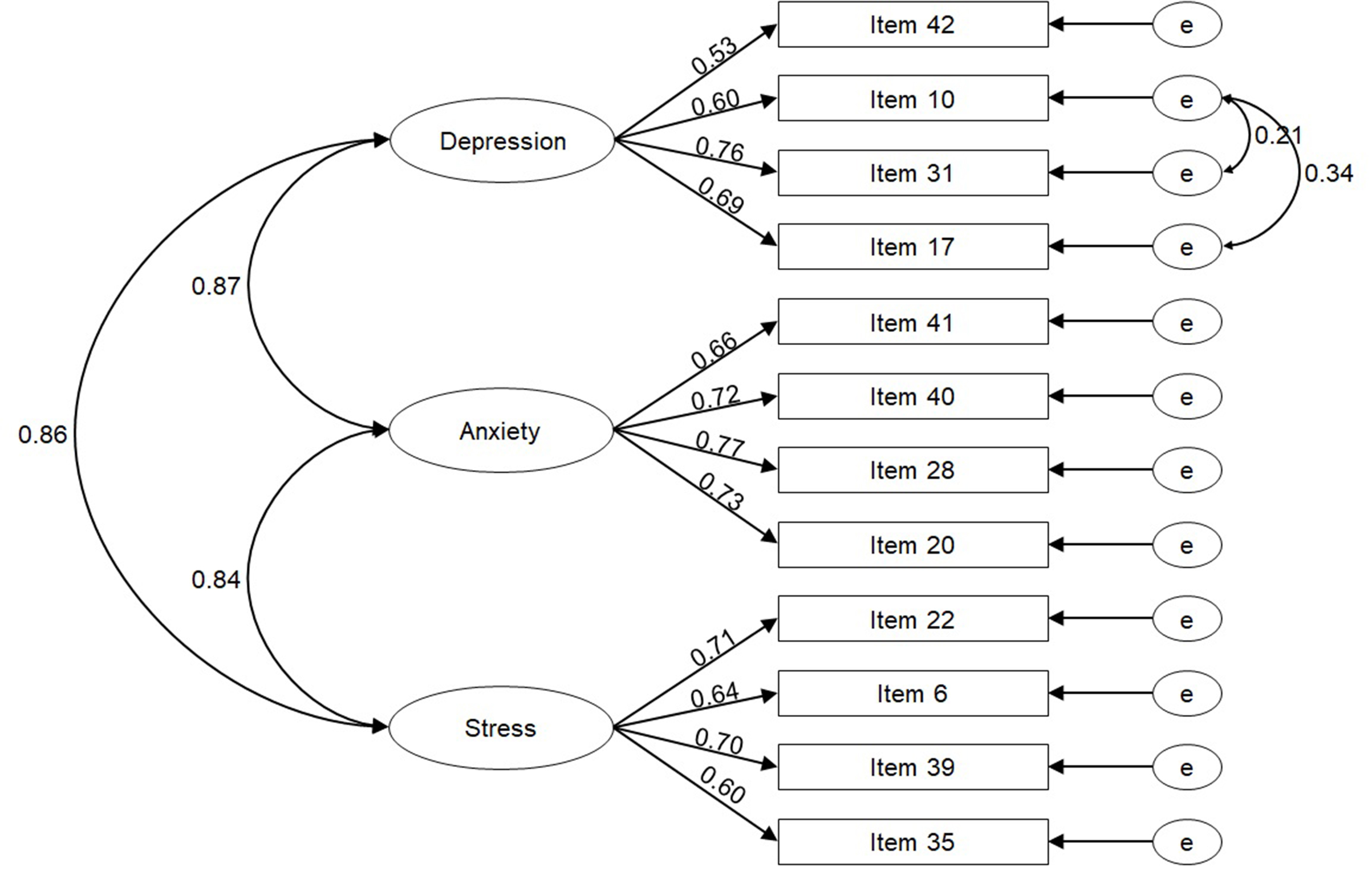

Supplement: Supplementary file 12 [file Image_9.JPG]

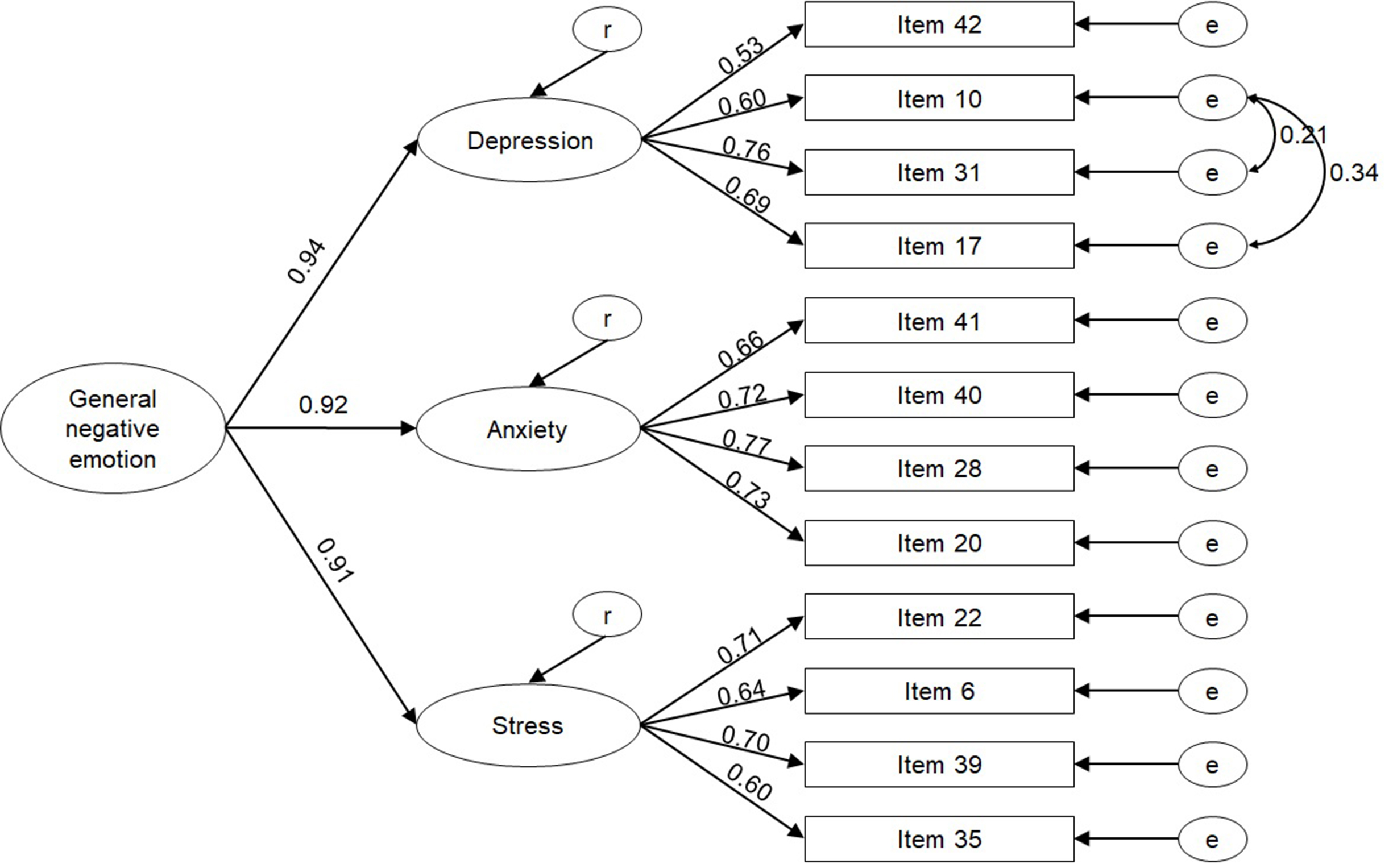

Supplement: Supplementary file 13 [file Image_10.JPG]
